# Preliminary Study of the Characterization of the Viable but Noncultivable State of *Yersinia enterocolitica* Induced by Chloride and UV Irradiation

**DOI:** 10.3390/microorganisms12091778

**Published:** 2024-08-28

**Authors:** Xueyu Hu, Xiaoxu Wang, Honglin Ren, Chengwei Li, Bo Zhang, Ruoran Shi, Yuzhu Wang, Shiying Lu, Yansong Li, Qiang Lu, Zengshan Liu, Pan Hu

**Affiliations:** 1 State Key Laboratory for Diagnosis and Treatment of Severe Zoonotic Infectious Diseases, Key Laboratory for Zoonosis Research of the Ministry of Education, Institute of Zoonosis, College of Veterinary Medicine, Jilin University, Changchun 130062, China; 2 Institute of Special Animal and Plant Sciences of Chinese Academy of Agricultural Sciences, Changchun 130112, China

**Keywords:** VBNC, *Yersinia enterocolitica*, UV, NaClO

## Abstract

The viable but non-culturable (VBNC) state is a survival strategy for many foodborne pathogens under adverse conditions. *Yersinia enterocolitica* (*Y. enterocolitica*) as a kind of primary foodborne pathogen, and it is crucial to investigate its survival strategies and potential risks in the food chain. In this study, the effectiveness of ultraviolet (UV) irradiation and chlorine treatment in disinfecting the foodborne pathogen *Y. enterocolitica* was investigated. The results indicated that both UV irradiation and chlorine treatment can induce the VBNC state in *Y. enterocolitica*. The bacteria completely lost culturability after being treated with 25 mg/L of NaClO for 30 min and a UV dose of 100 mJ/cm². The number of culturable and viable cells were detected using plate counting and a combination of fluorescein and propidium iodide (live/dead cells). Further research found that these VBNC cells exhibited reduced intracellular Adenosine Triphosphate (ATP) levels, and increased levels of reactive oxygen species (ROS) compared to non-induced cells. Morphologically, the cells changed from a rod shape to a shorter, coccobacillary shape with small vacuoles forming at the edges, indicating structural changes. Both condition-induced VBNC-state cells were able to resuscitate in tryptic soy broth (TSB) medium supplemented with Tween 80, sodium pyruvate, and glucose. These findings contribute to a better understanding of the survival mechanisms of *Y. enterocolitica* in the environment and are of significant importance for the development of effective disinfection strategies.

## 1. Introduction

The VBNC state is a critical survival strategy for bacteria under adverse environmental conditions. Many foodborne pathogens are induced into the VBNC state when faced with stressors such as *vibrio vulnificus*, which could enter the VBNC state in the low temperatures [1], *Escherichia coli* (*E. coli*) O157:H7, which could enter the VBNC state in the osmotic stress [2], oxidative stress, which could cause *E. coli* O157:H7 to enter the VBNC state [3], *Staphylococcus aureus* (*S. aureus*), which could enter the VBNC state in the nutritional imbalances [4], and UV radiation, which could cause *Pseudomonas aeruginosa* (*P. aeruginosa*) to enter the VBNC state [5]. Despite the inability of these bacteria to grow on standard culture media, they maintain certain metabolic activities and retain potential toxicity [6]. Traditional bacterial monitoring methods, which rely on the culturability of bacteria, make the detection of VBNC cells challenging, posing significant risks to food safety, water quality monitoring, and public health. Furthermore, bacteria in the VBNC state may revert to a cultivable state when exposed to favorable environmental conditions, regaining their ability to grow and reproduce, which could be accompanied by the recovery of their virulence and pathogenicity [7]. Concurrently, these pathogens’ resistance to stress is enhanced [8]. Therefore, effective monitoring and control of VBNC bacteria in food and water systems are essential to ensure safety and prevent potential outbreaks of foodborne and waterborne diseases.

*Y. enterocolitica* is a psychrotrophic Gram-negative bacillus that serves as a significant zoonotic pathogen and a leading cause of various human gastrointestinal diseases. According to a report from the European Union, yersiniosis, caused by this bacterium, has become the third most common zoonotic disease and is among the most critical foodborne zoonoses.In 2017, a report from the European Union stated that for every 100,000 individuals, 1.77 were infected with *Y. enterocolitica* [9]. Infections in infants are primarily characterized by diarrhea, while in adults, *Y. enterocolitica* can cause gastrointestinal diseases as well as respiratory and cardiovascular disorders. The main clinical symptoms of infection include diarrhea, acute enteritis, mesenteric lymphadenitis, and in severe cases, sepsis [10]. *Y. enterocolitica* is widely distributed in the natural environment and can contaminate a variety of food products, such as raw meat, dairy products, and vegetables, as well as being present in wildlife, water sources, and soil. Consumption of food and water are the primary routes for *Y. enterocolitica* infections [11]. Studies have shown that treatments with neutral electrolyzed water [12] and lactic acid [13] can induce *Y. enterocolitica* to enter the VBNC state, which enhances the bacterium’s persistence and survival in the environment and food products, posing significant challenges for food safety and public health management.

Disinfection is crucial for inactivating pathogens and preventing the spread of diseases. Chlorination and UV irradiation are widely used for the elimination of pathogenic microorganisms in various settings. The principle of chlorination disinfection involves the strong oxidizing properties of chlorine or its compounds (such as chlorine gas, bleaching powder, and bleaching powder concentrate) to penetrate bacterial cell walls and disrupt their internal enzyme systems, leading to bacterial death [14]. Chlorination is primarily used for the treatment of drinking water, domestic sewage, and industrial wastewater, as well as for the disinfection of swimming pool water [15]. In the food processing industry, chlorination is also employed for the disinfection of production water, equipment surfaces, and packaging materials [16]. UV disinfection mainly relies on UV-C light (with wavelengths between 200–300 nanometers) to damage the DNA or RNA structures of microorganisms (such as bacteria, viruses, fungi, and algae) [17]. This damage prevents microorganisms from replicating and reproducing, thereby achieving a bactericidal effect. UV disinfection does not introduce chemical substances during the process, so it does not produce disinfection by-products, making it widely applied in drinking water treatment, wastewater treatment, disinfection of medical devices, air disinfection, and microbial control in the food and beverage industry [18]. In water treatment, UV disinfection can effectively inactivate pathogens that are difficult to kill with traditional chemical disinfection methods, such as Cryptosporidium and Giardia [19].

UV disinfection and chlorination are widely employed physical and chemical methods for reducing the presence of various pathogenic microorganisms. As research advances and technologies for assessing bacterial viability improve, studies have increasingly shown that these disinfection techniques may not completely eradicate some bacteria, but instead, induce them into a VBNC state. For example, research has demonstrated that *E. coli*, isolated from hospital wastewater, can remain viable after chlorination, even when it is no longer culturable [20]. Similarly, *P. aeruginosa* have been induced into the VBNC state following treatment with 4 mg/L chlorine for 90 min [21]. Both *E. coli* and *P. aeruginosa* can also enter the VBNC state under UV disinfection [5], and a range of UV doses from 5 mJ/cm^2^ to 200 mJ/cm^2^ have been shown to induce VBNC states in *Acinetobacter* and *S. aureus*, with these bacteria maintaining some metabolic activities [22]. While certain physical or chemical disinfection methods are known to induce the VBNC state in *Y. enterocolitica*, there is a lack of research on whether the commonly used disinfection methods of chlorination and UV radiation can induce this state in this bacterium.

This study primarily explores the effectiveness of two common disinfection methods—chlorination and UV irradiation—in their ability to reduce the number of culturable bacteria and to induce the VBNC state. The research assesses the morphological changes, such as cell size and shape, in cells exposed to these treatments and determines the biological characteristics, such as ATP and ROS level and differences of *Y. enterocolitica* when in the VBNC state as induced by these methods. Additionally, the study investigates the potential for resuscitation of the bacteria in this state.

## 2. Materials and Method

### 2.1. Bacterial Strain and Culture Conditions

In this experiment, *Y. enterocolitica* CMCC52225, along with two isolates (5-3, L1+F 7-1) obtained from meat and contaminated water sources, were used. All *Y. enterocolitica* strains were stored in our laboratory and frozen at −80 °C. They were then inoculated onto tryptic soy agar plates (TSA; Land Bridge Technology, Beijing, China) and cultured at 28℃ for 24 h to reach an active state. Subsequently, the culture was inoculated into (Land Bridge Technology, Beijing, China) and incubated at 28 °C at 150 rpm on a shaker for 18 h until the logarithmic phase was reached. A sodium hypochlorite solution containing 10% available chlorine (Tianli Chemical Reagent Co. Ltd., Tianjin, China) was diluted with sterile deionized water (DW) to a working solution with a free available chlorine concentration of 5 mg/mL for use in subsequent experiments. A 0.5% solution of sodium thiosulfate (Xilong Scientific Co., Ltd., Shantou, China) was prepared and filtered through a 0.2 μm pore filter.

### 2.2. MIC and MBC Values

The bacterial suspension was diluted by sterile saline solution. Dissolved sodium hypochlorite (NaClO) in TSB to achieve final concentrations of 100, 80, 50, 40, 25, 20, 12.5, and 10 mg/L. Distributed these concentrations evenly in a 96-well transparent microplate. Measured OD_600_ in each well at 0 and 24 h. The NaClO concentration corresponding to an OD difference of less than 0.05 was defined as the Minimum Inhibitory Concentration (MIC). Incubated samples with MIC and higher concentrations in a sealed environment at 28 °C for 24 h. Afterwards, removed the plates and observed: the lowest concentration showing no visible bacterial colonies was identified as the Minimum Bactericidal Concentration (MBC). Performed each experiment in triplicate to ensure reliability of results.

### 2.3. Induction of VBNC State Y. enterocolitica by Sodium Hypochlorite and UV

The MIC of Sodium hypochlorite against *Y. enterocolitica* CMCC52225 and the isolates was 25 mg/L. The MBC of Sodium hypochlorite against *Y. enterocolitica* CMCC52225 and the isolates was 50 mg/L.

Cells were centrifuged from TSB, collected, and resuspended in 10 mL of distilled water containing sodium hypochlorite to form an induction system, to achieve a bacterial density of 10^8^ CFU/mL (Colony-FormingUnits/mL refers to the quantity of microbial cells in a 1-mL liquid sample that are capable of growing and forming visible colonies in the agar plates) and an effective chlorine concentration of 25 mg/L. The induction system was incubated in the dark at 28 °C with 120 rpm. At 0, 5, 10, 15, 20, 25, 30, 35, and 40 min, 2 mL was taken and sodium thiosulfate was added to terminate the reaction. After two rounds of centrifugation and washing with an equal volume of physiological saline, the bacteria were resuspended with 200 μL and spread onto TSA solid agar. After incubation at 28 °C for 24 h, the total bacterial count was recorded. When the number of culturable cells was less than 1 CFU/mL, the bacterial suspension used for plating was concentrated 10-fold and recounted. When the concentration of culturable cells was less than 0.1 CFU/mL, it indicated that all cells are non-culturable. UV induction was performed in 75-mm glass petri dishes, where cells were collected from TSB and resuspended in 20 mL of sterile physiological saline in the glass dish, achieving a final bacterial density of 10^7^ CFU/mL. The glass petri dish was placed on a magnetic stirrer for continuous stirring (120 rpm) and exposed to UV light, the liquid level kept 37 cm below the UV lamp (25 W, 254 nm, Philips, Andover, MA, USA). The UV radiation intensity was measured by a UV radiometer (UV-B, HANDY, Shanghai, China). Samples were collected at specific exposure times and spread onto TSA solid agar (Land Bridge Technology, Beijing, China). All samples were incubated at 28 °C for 24 h. The number of culturable cells was recorded after incubation. All experiments were performed in triplicate.

### 2.4. Cells Viability Assessment and Membrane Integrity Investigating

To evaluate the cell viability of *Y. enterocolitica* induced by sodium hypochlorite and UV, we assessed bacterial activity with the 5-cyano-2,3-ditolyl tetrazolium chloride (CTC) staining. A total of 3.5 milligrams of CTC were dissolved in 1 mL of ddH2O to prepare a stock solution with a concentration of 10 mM. We added 100 μL of the CTC stock solution to 100 μL of bacterial suspension to achieve a final concentration of 5 mM. Samples were incubated at 37 °C for 2 h in the dark, and 10 μL of the stained suspension was transferred onto a glass slide then observed on a Confocal laser scanning microscope (Olympus FV1000, Tokyo, Japan) at a magnification of 1000×. The excitation wavelength was set at 480 nm, and the emission wavelength was set at 560 nm. Cells with respiratory activity emit red fluorescence. In order to investigate the variations in cell membrane integrity, we used the LIVE/DEAD BacLight kit from Thermo Fisher Scientific, Waltham, MA, USA. Following the manufacturer’s protocol, we stained the cells with a mixture of SYTO 9 and PI. Specifically, 3 μL of the SYTO 9 and PI mixture were added to 1 mL of cell suspension. After being incubated at 25 °C for 15 min, 10 μL of the stained suspension was transferred onto a glass slide. Cells were observed using a Confocal laser scanning microscope at a magnification of 1000×. The excitation wavelength was set at 488 nm, and the emission wavelength was set at 561 nm. Green fluorescence indicates that the cell membrane is intact, while red fluorescence signifies cell membrane damage.

### 2.5. Counting VBNC Cells

Flow cytometry was used to count cells in different states. Initially, the bacterial was adjusted to 10^6^ CFU/mL. We used the LIVE/DEAD BacLight bacterial viability kit with SYTO 9 and PI dyes to record the number of cells with an intact cell membrane and the number of cells with a compromised cell membrane. We used the CTC staining method to label live cells. According to the user manual, we prepared 0.2 mL of sample for flow cytometry analysis. All samples were examined by the BD FACSVerse flow cytometer (BD Biosciences, Franklin Lakes, NJ, USA). All experiments were performed in triplicate.

### 2.6. Extracellular Protein and Intracellular Nucleic Acid Content Detection

After the induction under different conditions was completed, we centrifuged and collected the supernatant from the induction system. We then measured the extracellular protein concentration of the induction and non-induction *Y. enterocolitica* using the BCA Protein Assay Kit (Solarbio, Beijing, China). Then, we calculated the protein concentration of each protein sample according to the standard curve.

The DNA of the cells treated with UV or sodium hypochlorite was extracted by a DNA extraction kit (TIANGEN, Beijing, China). Then, we separated the DNA products by 1% agarose gel electrophoresis, stained them with a nucleic acid dye, and visualized them using the Bio-RAD imaging system (Bio-RAD, Hercules, CA, USA).

### 2.7. Morphological Analysis of VBNC Cells

After treatment with 25 mg/L chlorine for 30 min or 100 mJ/cm^2^ UV radiation, *Y. enterocolitica* was induced into the VBNC state. Additionally, high doses of chlorine and UV radiation were used to completely kill the bacteria. The cells were centrifuged and fixed with a 2.5% glutaraldehyde solution at 4 °C for 24 h. Morphological differences between VBNC cells under various induction conditions and non-induced *Y. enterocolitica* were observed by a scanning electron microscope (SEM, Hitachi S-3400N, Tokyo, Japan) and a transmission electron microscope (TEM, Hitachi H-7650, Japan).

### 2.8. Determination of the ATP Level

Leveraging the principle that luciferase catalyzes the luminescence of luciferin in the presence of ATP, we used the BacTiter-Lumi™ Luminescent Microbial Cell Viability Assay Kit (Beyotime Biotechnology, Shanghai, China) to measure the intracellular ATP levels. This kit relies on the ATP-dependent luminescence reaction catalyzed by luciferase, allowing ATP quantification through the measurement of chemiluminescence intensity. Before the assay, dead bacteria were introduced into the untreated live bacterial sample to establish a baseline for live bacterial proportion. Following the manufacturer’s protocol, bacterial suspension was mixed with an equal volume of BacTiter-Lumi™ detection reagent, and the resulting luminescence was quantified using a multimode microplate reader (TECAN/Spark; Molecular Devices, San Jose, CA, USA). The experiment was independently repeated three times. The levels of ATP were expressed as relative fluorescence intensity across the various samples in this study.

### 2.9. Determination of the Level of Intracellular ROS

A ROS Assay Kit was used to detect the levels of ROS. Cells in the VBNC state induced by chlorine or UV were separated using a density gradient centrifugation method to reduce the proportion of dead bacteria, and the bacteria were then resuspended in 60% Percoll. Subsequently, the suspension was centrifuged at 4 °C and 12,000× *g* for 40 min. Finally, the viable cell suspension was obtained from the middle and bottom layers of the Percoll gradient. Non-induced cells, cells completely killed by sodium hypochlorite or UV, and cells were resuspended in PBS to 10^7^ CFU/mL. The fluorescent probe DCFH-DA (2′,7′-dichlorofluorescein diacetate; Beyotime Biotechnology, Shanghai, China) was added to the bacterial suspension in a black 96-well plate and incubated in a 37 °C incubator for 20 min. Fluorescence was measured with excitation and emission wavelengths of 488 nm and 525 nm, respectively. The experiment was independently repeated three times. The levels of ROS were measured in terms of relative fluorescence intensity.

### 2.10. Resuscitation of the VBNC State of Y. enterocolitica

The resuscitation capacity assay experiment was performed with reference to the method of Cheng [13] with slight modifications. In this study, we used four distinct resuscitation media to assess the resuscitation capacity of *Y. enterocolitica* under VBNC conditions. Cells that were induced into the VBNC state using 25 mg/L NaClO and UV radiation, as well as Control cells that were not subjected to any induction or treatment, were centrifuged and washed with physiological saline. To minimize the potential influence of any remaining culturable bacteria on the results, all samples for resuscitation were serially diluted twice. Furthermore, in addition to the Control group, another Control group, designated as the Treatment group, was established. This group adopted the method of continuously diluting the cells in the logarithmic growth phase to 10^2^ CFU/mL to simulate the growth curve of viable bacteria that might have been missed in detection. The bacterial suspensions were then incubated in media containing 5% (*v*/*v*) Tween 80-TSB, 2% glucose-TSB, and 2 mg/mL sodium pyruvate-TSB, alongside the TSB group. The samples were placed at 28 °C and cultured for 48 h; then, the optical density at 600 nm was measured for each well at regular intervals. Before the experiments, dead bacteria were introduced into Control cells and VBNC cells for resuscitation experiments to ensure that the number of viable bacteria in the same volume remained consistent. Each experiment was independently repeated three times to ensure consistency and reliability of the results.

### 2.11. Virulence Assay

We fed the *Caenorhabditis elegans* with *Y. enterocolitica* in different states instead of the *E. coli* OP50 to verify the changes in toxicity of *Y. enterocolitica* in different states. The nematode growth medium (NGM) agar plates were prepared following standard protocols [23]. *Caenorhabditis elegans* were fed with *E. coli* OP50 and cultured at 22 °C until a significant number of adults were observed on the plates. The worms were then synchronized by lysing the worms containing eggs to obtain the eggs. When the synchronized worms grew to the fourth (L4) larval stage, 20 larvae were transferred to a new NGM plate containing 5-Fluorouracil (MCE, Zelienople, PA, USA). Different plates were spread with a layer of *E. coli* OP50, culturable *Y. enterocolitica*, or *Y. enterocolitica* in the VBNC state, respectively. Subsequently, all the inoculations were incubated at 22 °C and observed daily. When *Caenorhabditis elegans* do not respond to touch, they are considered dead.

### 2.12. Statistical Analysis

Data were presented as means ± SD. Statistical significance was calculated with GraphPad Prism 7.0 (GraphPad Software Inc., San Diego, CA, USA) using the multiple *t*-tests. Figures were produced by GraphPad Prism 7.0.

## 3. Results

### 3.1. Culturability of Y. enterocolitica

**Figure 1:**
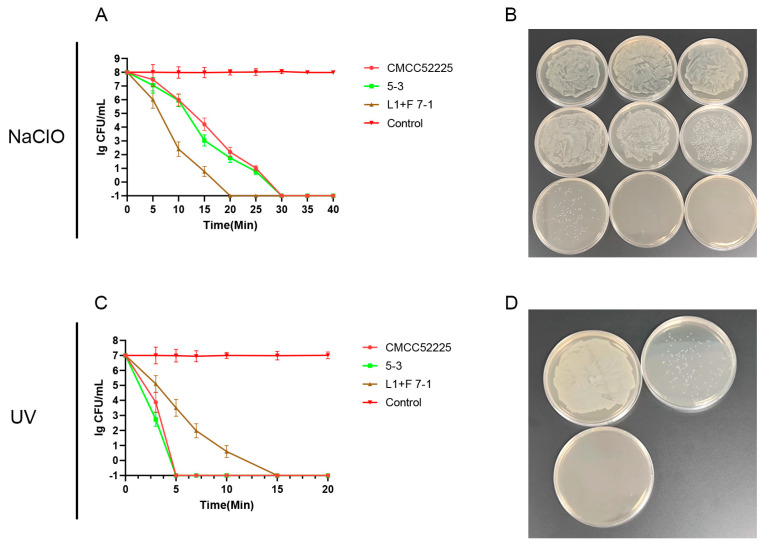
The culturable colonies counts for *Y. enterocolitica* after treatment with NaCIO with different treatment times (**A**); plate growth of *Y. enterocolitica* CMCC5225 after treatment with NaCIO, from left to right, are 0 min, 5 min, 10 min, 15 min, 20 min, 25 min, 30 min, 35 min, and 40 min, in sequence (**B**); the culturable colonies counts for *Y. enterocolitica* after treatment with UV with different treatment times (**C**); plate growth of *Y. enterocolitica* CMCC5225 after treatment with UV, from left to right, are 0 min, 3 min, and 5 min (**D**).

As illustrated in Figure 1, the culturability of *Y. enterocolitica* in distilled water showed no significant change within 40 min (approximately 10^8^ CFU/mL, *p* > 0.05). The culturability of *Y. enterocolitica* exposed to a solution containing sodium hypochlorite (25 mg/L) significantly decreased within 15 min, with the isolate L1+F 7-1 completely losing culturability after 20 min of exposure (<0.1 CFU/mL). *Y. enterocolitica* CMCC52225 and isolate 5-3 completely lost culturability after 30 min (<0.1 CFU/mL). UV radiation effectively reduced the culturability of *Y. enterocolitica*. At a dose of 100 mJ/cm^2^, isolate 5-3 and CMCC52225 completely lost culturability, while at a dose of 300 mJ/cm^2^, isolate L1+F 7-1 completely lost culturability.

### 3.2. Activity Detection of Y. enterocolitica

**Figure 2:**
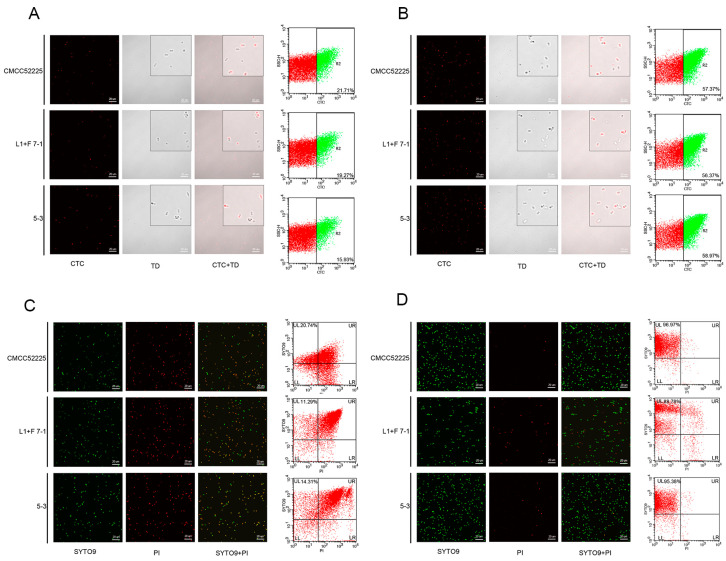
Confocal images of *Y. enterocolitica* and flow cytometric analysis of nonculturable cells at the point in time when cultivability was completely lost. Viable cells stained with CTC appear as red, R2: viable cells with intact membrane stained by CTC (**A**,**B**). Bacteria with intact membrane stained with SYTO 9 appear as green, bacteria with damaged membrane stained with PI appear as red. The subpopulations were classified based on their differential staining characteristics: UL: viable cells with intact membrane stained by SYTO9; UR: damaged cells stained by SYTO9 and PI; LR: dead cells stained with PI; LL: unstained cells (**C**,**D**).

Although the cells in all samples were non-culturable, some cells were still observed to emit red fluorescence under a Confocal laser scanning microscope. After CTC was absorbed by healthy living cells, it was reduced to CTC formazan that emitted red fluorescence through electron transport during respiratory activity; however, non-breathing cells failed to produce CTC formazan, which did not emit red fluorescence. Further analysis by flow cytometry indicated that after treatment with 25 mg/L sodium hypochlorite, which resulted in the loss of culturability, the viable cell ratios of *Y. enterocolitica* CMCC52225, isolate strains L1+F 7-1 and 5-3 were 21.71%, 19.27%, and 15.93%, respectively (Figure 2A). After treatment with UV light, which resulted in the loss of culturability, the viable cell ratios of *Y. enterocolitica* CMCC52225, isolate strains L1+F 7-1 and 5-3 were 57.37%, 56.37%, and 58.97%, respectively (Figure 2B). In this study, both 25 mg/mL sodium hypochlorite and UV irradiation induced the VBNC state in *Y. Enterocolitica,* because all samples were non-culturable, but some cells still survived. The proportion of *Y. enterocolitica* induced into the VBNC state by UV irradiation was significantly higher (*p* < 0.05) than the proportion induced by sodium hypochlorite.

### 3.3. Membrane Integrity Assay of Y. enterocolitica

As shown in Figure 2, after staining with SYTO 9 and PI, some cells with intact plasma membranes emitted green fluorescence, while cells with damaged plasma membranes emitted red fluorescence under the Confocal laser scanning microscope. Further analysis by flow cytometry revealed that after treatment with 25 mg/L sodium hypochlorite, the percentages of cells with intact plasma membranes in *Y. enterocolitica* CMCC52225, isolate L1+F 7-1, and isolate 5-3 were 20.74%, 11.29%, and 14.31%, respectively. After treatment with UV, the percentages of cells with intact plasma membranes in *Y. enterocolitica* CMCC52225, isolate L1+F 7-1, and isolate 5-3 were 96.97%, 88.78%, and 95.38%, respectively. Therefore, *Y. enterocolitica* can still maintain good integrity of its cell membranes after UV irradiation.

### 3.4. Extracellular Protein and Intracellular Nucleic Acid Content Detection

**Figure 3:**
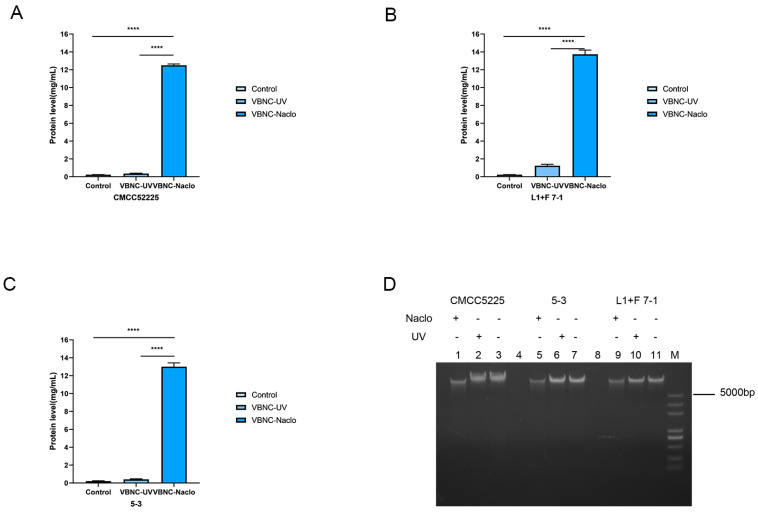
Intracellular protein leakage content after NaCIO or UV treatment (**A**–**C**), Effects of NaCIO or UV on the genomic DNA of *Y. enterocolitica* (**D**). **** indicates statistical significance at *p* < 0.001.

Damage to the cell membrane can lead to leakage of intracellular proteins, and excessive leakage can lead to bacterial death. After NaCIO treatment for 30 min, the leakage of intracellular proteins in *Y. enterocolitica* CMCC52225, isolate L1+F 7-1, and isolate 5-3 were 12.34 mg/mL, 13.76 mg/mL, and 13.16 mg/mL, respectively. The leakage of intracellular proteins in *Y. enterocolitica* CMCC52225, isolate L1+F 7-1, and isolate 5-3 were 0.43 mg/mL, 1.41 mg/mL, and 0.49 mg/mL, respectively, after treatment with UV, indicating that UV treatment has a relatively small effect on the leakage of intracellular proteins in *Y. enterocolitica* (Figure 3A–C). The destruction of the cell membrane and the resulting changes in permeability typically cause a significant release of proteins, thereby providing further validation for the aforementioned findings.

**Figure 4:**
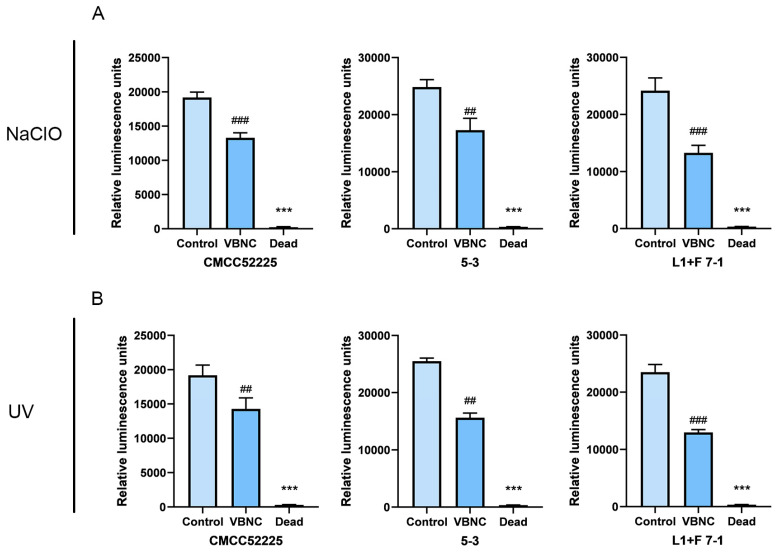
NaCIO (**A**) and UV (**B**) induced changes in intracellular ATP concentration in the VBNC state of *Y. enterocolitica*. Mean values of triplicate independent experiments and their SD are shown. ### and ## indicates statistical significance at *p* < 0.001 and *p* < 0.01 compared with the Control, *** indicates statistical significance at *p* < 0.001 compared with the VBNC.

DNA is the primary carrier of genetic information in organisms and is important for the survival and reproduction of organisms. Figure 4 shows the impact of NaCIO and UV on the level of intracellular nucleic acid of *Y. enterocolitica*. There was a significant difference in the DNA bands between the NaCIO treatment group and the untreated group. In Figure 3D, the brightness and clarity of the DNA in lanes 1, 5, and 9 were significantly weaker than those in lanes 3, 7, and 11. The DNA bands of the bacterial genomic DNA after UV treatment were not significantly different from those of the untreated bacterial genomic DNA. However, when exposed to NaCIO, the degree of DNA leakage in *Y. enterocolitica* was higher compared to bacteria treated with UV.

### 3.5. Intracellular ATP Level of VBNC State of Y. enterocolitica

The relative ATP content in the VBNC state induced by sodium hypochlorite and UV, as well as in live bacteria, was compared here through the intensity of light emission, since the ATP content is directly proportional to the luminescence intensity. As shown in Figure 4, the luminescence intensity of non-induced cells of *Y. enterocolitica* CMCC52225, isolate L1+F 7-1, and 5-3 were significantly higher than that of the VBNC-state cells induced by chlorine (Figure 4A) and UV (Figure 4B). Additionally, the luminescence intensity of dead cells was almost undetectable. This indicates that although the ATP level of cells in the VBNC state has decreased significantly, it still maintains a relatively high level.

### 3.6. The Level of Intracellular ROS

**Figure 5:**
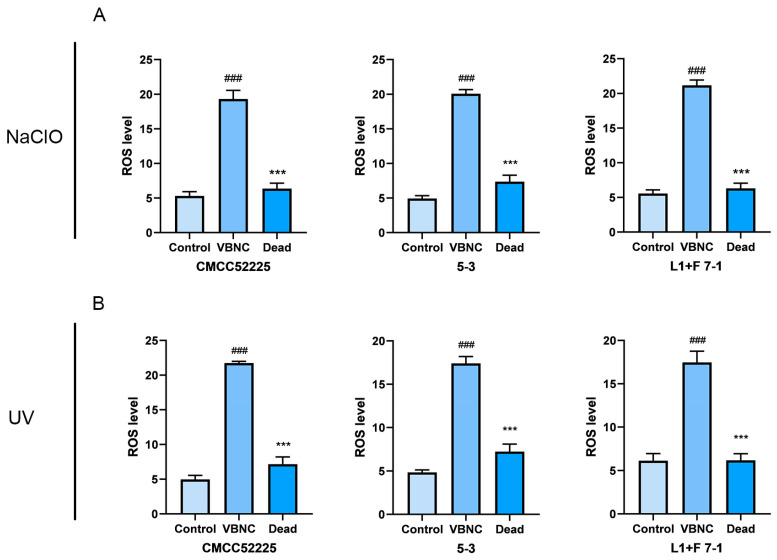
NaCIO (**A**) and UV (**B**) induced changes in intracellular ROS concentration in the VBNC state of *Y. enterocolitica*. Mean values of triplicate independent experiments and their SD are shown. ### indicates statistical significance at *p* < 0.001 compared with the Control, *** indicates statistical significance at *p* < 0.001 compared with the VBNC.

The intracellular ROS levels were measured in non-induced *Y. enterocolitica* and cells induced into the VBNC state by UV or sodium hypochlorite (Figure 5). After treatment with sodium hypochlorite, the ROS levels in *Y. enterocolitica* CMCC52225, isolate L1+F 7-1, and 5-3 were 19.32, 19.89, and 21.14, respectively. After treatment with UV, the ROS levels in *Y. enterocolitica* CMCC52225, isolates L1+F 7-1, and 5-3, were 21.82, 17.69, and 18.14, respectively. In non-induced cells of *Y. enterocolitica* CMCC52225, isolates L1+F 7-1 and 5-3, the ROS levels were 4.84, 4.91, and 5.82. Therefore, the intracellular ROS levels in VBNC-state cells were approximately five times higher than those in non-induced cells (*p* < 0.01).

### 3.7. Morphological Analysis of the VBNC-State Cells

**Figure 6:**
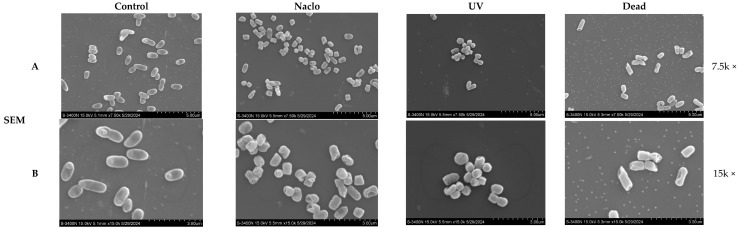

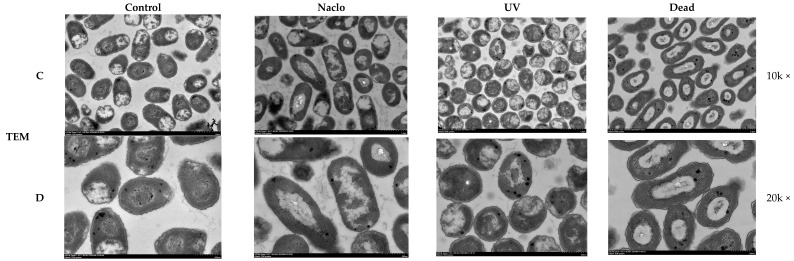
Electron microscopy observations of *Y. enterocolitica* CMCC52225. (**A**,**B**) SEM images of *Y. enterocolitica* CMCC52225 cells, the magnification of (**A**) is 7500, the magnification of (**B**) is 15,000; (**C**,**B**) TEM images of *Y. enterocolitica* CMCC52225 cells, the magnification of (**C**) is 10,000, the magnification of (**D**) is 20,000.

Culturable *Y. enterocolitica* cells were characterized by their rod or coccus shape, with intact and smooth surfaces, and exhibited a uniformly dense cytoplasm under electron microscopy. VBNC-state cells induced by low-level chlorine treatment (25 mg/L) showed partial preservation of the cell membrane, with some cells displaying reduced size and a less smooth surface compared to the Control cells. This observation indicated that chlorination disinfection impairs the cell membrane. In contrast, cells induced into the VBNC state by a low dose of UV radiation (100 mJ/cm^2^) maintained a more intact cell membrane, with only a slight reduction in size and a smoother surface observed in a few cells when compared to the Control. This suggests that UV disinfection does not affect the integrity of the cell membrane. In contrast, dead cells showed noticeable ruptures and severe wrinkling (Figure 6A,B).

Transmission electron microscopy (TEM) revealed that non-induced cells have a uniform distribution of cytoplasm and genetic material within the cell, with the cell membrane and cell wall remaining intact. In dead cells, large intracellular vacuoles are observed, and the area containing genetic material appears hollow. Cells in the VBNC state generally retained their structural integrity, with small vacuoles visible at the cell periphery, indicating damage to the genetic material (Figure 6C,D).

### 3.8. Resuscitation of VBNC-State Cells

**Figure 7:**
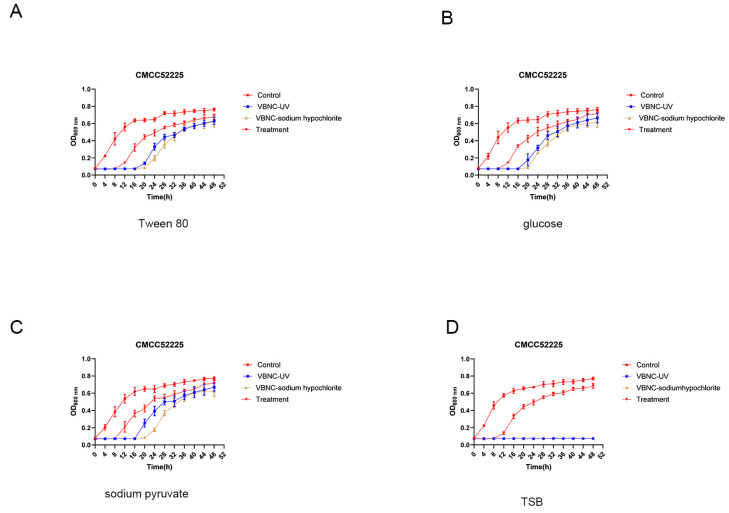
VBNC state *Y. enterocolitica* CMCC52225 induced by NaCIO and UV recovery in Tween 80-TSB (**A**), glucose-TSB (**B**), sodium pyruvate-TSB (**C**), and TSB (**D**).

In three types of resuscitation media—Tween80-TSB (Figure 7A), glucose-TSB (Figure 7B), and sodium pyruvate-TSB (Figure 7C)—non-induced cells were able to grow normally. In these three resuscitation media, VBNC state *Y. enterocolitica* CMCC52225 cells induced by chlorine were recovered after 20 to 24 h. VBNC-state cells induced by UV were resuscitated after 16 to 20 h, indicating that the VBNC-state cells induced by UV are more easily resuscitated, but all VBNC bacteria failed to revive in TSB. VBNC state *Y. enterocolitica* 5-3 (Supplementary Figure S1) and VBNC state *Y. enterocolitica* L1+F 7-1 (Supplementary Figure S2) have similar resuscitation capabilities.

### 3.9. Virulence Assay

**Figure 8:**
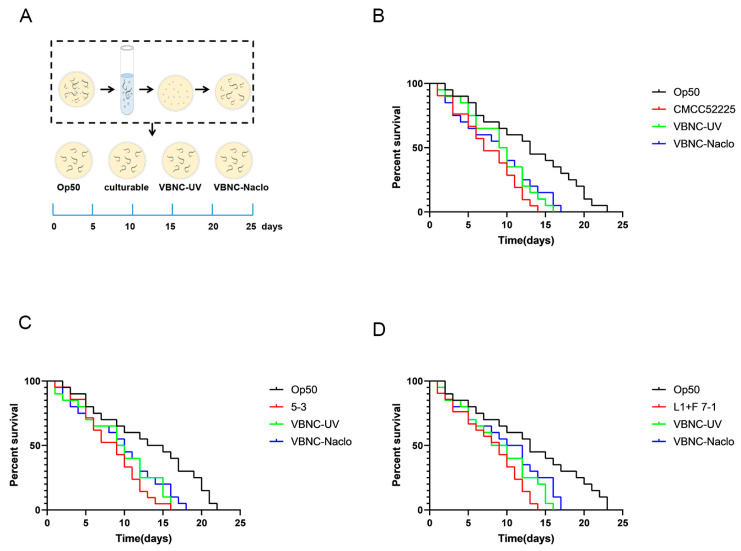
The pathogenicity of *Y. enterocolitica* in *Caenorhabditis elegans* infection models. Scheme of the experimental protocol for the *Y. enterocolitica* infection model (**A**); Survival rates of *Caenorhabditis elegans* infection different states of *Y. enterocolitica* CMCC52225 (**B**); Survival rates of *Caenorhabditis elegans* infection different states of *Y. enterocolitica* 5-3 (**C**); Survival rates of *Caenorhabditis elegans* infection different states of *Y. enterocolitica* L1+F 7-1 (**D**).

As shown in Figure 8, the VBNC-state cells and normal cells are both toxic to *Caenorhabditis elegans*. When exposed to normal cells, all *Caenorhabditis elegans* died within about 15 days. When exposed to cells in the VBNC state, all *Caenorhabditis elegans* died within approximately 16 days.

## 4. Discussion

UV and chlorination are two primary disinfection methods widely used for sanitization in various settings. Although it has been established that UV and sodium hypochlorite possess high bactericidal efficacy, a mounting number of studies show that certain bacteria can enter the VBNC state to adapt to environmental changes when exposed to stress conditions such as UV or chlorination [24,25,26]. This study investigates the effects of UV and sodium hypochlorite on the induction of the VBNC state in *Y. enterocolitica* and its characteristics, in order to comprehensively assess the bactericidal effects of UV and chlorination on this bacterium. The results show that after exposure to UV and sodium hypochlorite, live cells still exist even after all *Y. enterocolitica* lose culturability, indicating that these cells are induced into the VBNC state by UV or chlorination.

Intracellular ATP levels are fundamental to cellular life processes and are among the most critical indicators for assessing cellular metabolic activity. As the results show, the relative concentration of intracellular ATP in cells induced into the VBNC state by UV and sodium hypochlorite is significantly lower than that in cells in the logarithmic growth phase, but still remains at a relatively high level. This is consistent with the findings of Meng et al. in their study on the stress response of *Escherichia coli* and *Staphylococcus aureus* to oligosaccharides [27]. The maintenance of a certain level of ATP metabolism suggests that VBNC-state cells still require energy to sustain basic physiological activities. This finding also contributes to the study of resuscitating VBNC-state cells. If the energy metabolism of VBNC-state cells is limited, increasing the supply of ATP can promote the recovery of key metabolic pathways within the cells, thus aiding in the resuscitation from the VBNC state, a conclusion proven by Yang and Wang [28]. Furthermore, it has been demonstrated that a decrease in ATP content can affect protein homeostasis, promote the formation of protein aggregates, and induce bacteria to enter the VBNC state [29]. Whether maintaining cellular energy metabolism can prevent bacteria from entering the VBNC state remains a hypothesis that requires further research to validate.

The impact of ROS on cells includes protein oxidation, DNA damage, and lipid peroxidation, thereby reducing the survival capacity of cells [30]. The results of this study show that the levels of ROS within cells induced into the VBNC state *Y. enterocolitica* by UV and sodium hypochlorite are significantly higher than the ROS levels within non-induced cells. This is consistent with the results of the existing studies. Ma and Xu et al. found that the absence of the transcriptional regulator RpoS increases the level of intracellular ROS, making Salmonella more prone to enter the VBNC state under low temperature or dry conditions [31]. Masmoudi et al. discovered that the deletion of katA or sodA genes leads to the loss of culturability in *Staphylococcus aureus* and activates the VBNC state [32]. Therefore, the increase in intracellular ROS levels may be an important reason for the maintenance of the VBNC state in *Staphylococcus aureus* induced by UV and sodium hypochlorite. Overall, the relationship between ROS and the VBNC state is complex. On one hand, the accumulation of ROS may lead bacteria to enter the VBNC state; on the other hand, the VBNC state may enable bacteria to better tolerate oxidative stress. Research into this relationship helps us to better understand how bacteria adapt to and survive in changing environmental conditions, which is of significant importance for the development of new antimicrobial strategies and the assessment of bacterial survival states in the environment.

By observing cellular morphology, differences between cells induced into the VBNC state by UV and sodium hypochlorite and non-induced cells can be directly discerned. Confocal electron microscopy results indicate that sodium hypochlorite disrupts the bacterial cell membrane, while the cell membrane structure remains intact under low-dose UV treatment, consistent with Zhu’s findings on the mechanisms of VBNC formation in *Escherichia coli* isolated from hospital wastewater following chlorination and UV disinfection [20]. The reason is mainly related to the principle of UV disinfection and sodium hypochlorite disinfection. UV light of the appropriate wavelength can penetrate the cell membrane directly, damaging the genetic material within microbial cells, thereby killing the bacteria. The disruption of the DNA or RNA structure by UV light causes bacteria to lose their ability to replicate and grow. Additionally, as the duration of UV radiation increases, a significant amount of ozone may form in the environment. The strong oxidizing effect of ozone can further damage the cell membrane, which is consistent with the phenomena observed in subsequent experiments. The strong oxidizing effect of hypochlorite often damages the cell membrane directly. Scanning electron microscopy reveals that cells induced into the VBNC state by UV and sodium hypochlorite have roughened surfaces but still maintain a spherical shape. Studies on the VBNC state of *E. coli* have found that E. coli in the VBNC state have roughened cell surfaces, with cell shapes changing to curved, shorter rods, and reduced in size [33,34], which is consistent with our result. Nan et al. propose that cells reduce in size as a strategy to lower their maintenance needs. They also suggest that smaller cell formations offer protection to non-spore-forming bacteria in harsh environmental conditions [35]. According to transmission electron microscopy results, in non-induced cells, the cytoplasm and nucleic material were divided evenly. Ribosomes are well-distributed throughout the cytoplasmic matrix, and the nucleoid is concentrated at the center of the cell. Additionally, the cell membrane and cell wall are closely aligned. With the onset of the VBNC state, significant changes occur in the internal structure of the cells. Compared to non-induced cells, the electron density of the cytoplasm increases in VBNC cells, and there is a gap between the cell membrane and cell wall that is visible in some VBNC cells. This gap may be due to cytoplasmic condensation, leading to cell wall deformation and irregular cell morphology in VBNC cells. Cheng et al. showed similar results in the study of *Y. enterocolitica* VBNC state induced by lactic acid stress. The VBNC-state cells induced by lactic acid changed from long rod-like to short rod-like, with small vacuoles at the cell edges; the genetic material was loosened, and the density of cytoplasm was increased [13]. Bai et al. also point out that cytoplasmic condensation leads to an increase in ribosome concentration, which in turn causes cytoplasmic crowding and significantly reduces the freedom of molecular motion, thereby decreasing cellular metabolic activity and enabling VBNC-state cells to adapt to harsh external environments [36]. The condensation of the cytoplasm and the increase in ribosome concentration in VBNC cells may be a survival strategy adopted by cells to cope with unfavorable environmental conditions.

Resuscitation refers to the process by which VBNC-state cells regain cultivability, indicating that the cells’ physiological and metabolic processes return to normal levels, and they also regain their virulence, posing a potential threat. There are currently two controversies regarding resuscitation: one suggests that VBNC-state cells in a favorable environment recover their normal physiological and metabolic activities [37], while the other posits that resuscitation occurs due to the division of undetected cultivable cells [38]. In this study, we used four resuscitation conditions, including sodium pyruvate-TSB, Tween 80-TSB, glucose-TSB, and TSB. Additionally, we diluted the VBNC state bacterial suspension to reduce the chances of undetected cultivable cells participating in the resuscitation process, and we recorded the growth curves of low numbers of cultivable bacteria to effectively distinguish between true resuscitation of VBNC-state cells and division of cultivable cells. The results showed that VBNC-state cells induced by UV and sodium hypochlorite, even when diluted 100-fold (culturable cell count < 0.001 CFU/mL), were able to undergo resuscitation under certain conditions, and the growth curves of low numbers of cultivable bacteria exhibited inflection points earlier than the resuscitation curves. Low quantities of culturable bacteria in the early stages of growth have a slow increase in bacterial concentration due to a smaller number of cell divisions; this kind of increase cannot be reflected by changes in the OD (Optical Density) values detected by an enzyme-linked immunosorbent assay (ELISA) reader. However, as the bacteria grow exponentially, the number of cells dividing increases, leading to a significant rise in bacterial concentration, at which point the ELISA reader can detect the inflection point in the growth curve. In the recovery experiment for the Control group of cells and VBNC cell samples, despite containing the same number of viable bacteria, the VBNC cell recovery group had an undetectable concentration change for a long period before the concentration showed a significant change that could be detected by the ELISA reader. Moreover, the time for the VBNC cell recovery group to show a significant change in concentration was longer than the time for the low number of culturable bacteria to show a significant increase in concentration. This indicates that the significant change in the VBNC cell recovery group is due to the resuscitation of VBNC cells. At the same time, there was no significant change in the concentration of VBNC cells in TSB, while cultivable cells grew normally in TSB. These results indicate that *Y. enterocolitica* cells in the VBNC state induced by UV and sodium hypochlorite indeed underwent resuscitation. Although both UV and sodium hypochlorite-induced VBNC state *Y. enterocolitica* cells underwent resuscitation, those induced by UV seemed easier to resuscitate, which may be related to the more intact preservation of their cell membrane structure. This is consistent with Hu’s findings on the resuscitation of VBNC state Acinetobacter, where extended induction times with chlorine led to increased cell membrane damage and prolonged resuscitation times [39]. Additionally, the toxicity analysis of VBNC-state *Y. enterocolitica* revealed that the VBNC state remains toxic to *Caenorhabditis elegans*, indicating that its high-risk potential in public health is not only due to its ability to resuscitate.

## 5. Conclusions

In this study, it was demonstrated for the first time that both UV and sodium hypochlorite can induce various strains of *Y. enterocolitica* (including reference strains and isolates) into the VBNC state. Cells in the VBNC state could be resuscitated in glucose-TSB, 5% Tween 80-TSB, and 2 mg/mL sodium pyruvate-TSB, with cells induced into the VBNC state by UV showing a higher resuscitation rate. Compared to non-induced cells, VBNC-state cells exhibited reduced intracellular ATP concentration, while the intracellular ROS levels were elevated. Cells entering the VBNC state changed from a rod shape to a shorter rod shape, with small vacuoles at the cell edges; the genetic material became loose, and the cytoplasmic density increased. Low-dose UV treatment caused less damage to the cell membrane structure compared to sodium hypochlorite, making resuscitation easier. This finding may prompt new thoughts on the focus of disinfection strategies. In summary, both UV and sodium hypochlorite can induce *Y. enterocolitica* into the VBNC state, which is difficult to detect and poses a threat to food safety. Therefore, it is recommended to increase the dosage of disinfectants or enhance UV intensity and extend the disinfection time during the sanitization process.

## Supplementary Materials

The following supporting information can be downloaded at: https://www.mdpi.com/article/10.3390/microorganisms12091778/s1, Figure S1: VBNC state *Y. enterocolitica* 5-3 induced by Naclo and UV recovery in Tween 80-TSB (A), glucose-TSB (B) and sodium pyruvate-TSB (C) and TSB(D). Figure S2: VBNC state *Y. enterocolitica* L1+F 7-1 induced by Naclo and UV recovery in Tween 80-TSB (A), glucose-TSB (B) and sodium pyruvate-TSB (C) and TSB(D).

## References

[1] Abe A., Ohashi E., Ren H., Hayashi T., Endo H. (2007). Isolation and characterization of a cold-induced nonculturable suppression mutant of *Vibrio vulnificus*. Microbiol. Res..

[2] Li Y., Huang T.Y., Ye C., Chen L., Liang Y., Wang K., Liu J. (2020). Formation and control of the viable but non-culturable state of foodborne pathogen *Escherichia coli* O157:H7. Front. Microbiol..

[3] Lin H., Ye C., Chen S., Zhang S., Yu X. (2017). Viable but non-culturable *E. coli* induced by low level chlorination have higher Persistence to antibiotics than their culturable counterparts. Environ. Pollut..

[4] Pinto D., Santos M.A., Chambel L. (2015). Thirty years of viable but nonculturable state research: Unsolved molecular mechanisms. Crit. Rev. Microbiol..

[5] Zhang S., Ye C., Lin H., Lv L., Yu X. (2015). UV disinfection induces a VBNC state in *Escherichia coli* and Pseudomonas aeruginosa. Environ. Sci. Technol..

[6] Yin W., Yang L., Zhou X., Liu T., Zhang L., Xu Y., Li N., Chen J., Zhang Y. (2023). Peracetic acid disinfection induces antibiotic-resistant *E. coli* into VBNC state but ineffectively eliminates the transmission potential of ARGs. Water Res..

[7] Pinto D., Almeida V., Almeida Santos M., Chambel L. (2011). Resuscitation of *Escherichia coli* VBNC cells depends on a variety of environmental or chemical stimuli. J. Appl. Microbiol..

[8] Liu J., Yang L., Kjellerup B.V., Xu Z. (2023). Viable but nonculturable (VBNC) state, an underestimated and controversial microbial survival strategy. Trends Microbiol..

[9] Leon-Velarde C.G., Jun J.W., Skurnik M. (2019). *Yersinia* Phages and Food Safety. Viruses.

[10] Bottone E.J. (1999). Yersinia enterocolitica: Overview and epidemiologic correlates. Microbes Infect..

[11] Gupta V., Gulati P., Bhagat N., Dhar M.S., Virdi J.S. (2015). Detection of Yersinia enterocolitica in food: An overview. Eur. J. Clin. Microbiol. Infect. Dis. Off. Publ. Eur. Soc. Clin. Microbiol..

[12] Han D., Hung Y.C., Wang L. (2018). Evaluation of the antimicrobial efficacy of neutral electrolyzed water on pork products and the formation of viable but nonculturable (VBNC) pathogens. Food Microbiol..

[13] Cheng S., Li Z., Bai X., Feng J., Su R., Song L., Yang H., Zhan X., Xia X., Lü X. (2023). The biochemical characteristics of viable but nonculturable state Yersinia enterocolitica induced by lactic acid stress and its presence in food systems. Food Res. Int..

[14] Dodd M.C. (2012). Potential impacts of disinfection processes on elimination and deactivation of antibiotic resistance genes during water and wastewater treatment. J. Environ. Monit. JEM.

[15] Azuma T., Hayashi T. (2021). On-site chlorination responsible for effective disinfection of wastewater from hospital. Sci. Total Environ..

[16] Wang Y., Chen Z., Zhao F., Yang H. (2023). Metabolome shifts triggered by chlorine sanitisation induce *Escherichia coli* on fresh produce into the viable but nonculturable state. Food Res. Int..

[17] Ben Said M., Masahiro O., Hassen A. (2010). Detection of viable but non cultivable *Escherichia coli* after UV irradiation using a lytic Qβ phage. Ann. Microbiol..

[18] Bonetta S., Pignata C., Bonetta S., Amagliani G., Brandi G., Gilli G., Carraro E. (2021). Comparison of UV, Peracetic Acid and Sodium Hypochlorite Treatment in the Disinfection of Urban Wastewater. Pathogens.

[19] Belosevic M., Craik S.A., Stafford J.L., Neumann N.F., Kruithof J., Smith D.W. (2001). Studies on the resistance/reactivation of Giardia muris cysts and Cryptosporidium parvum oocysts exposed to medium-pressure ultraviolet radiation. FEMS Microbiol. Lett..

[20] Zhu L., Shuai X., Xu L., Sun Y., Lin Z., Zhou Z., Meng L., Chen H. (2022). Mechanisms underlying the effect of chlorination and UV disinfection on VBNC state *Escherichia coli* isolated from hospital wastewater. J. Hazard. Mater..

[21] Qi Z., Liu C. (2022). Metabolic characteristics and markers in viable but nonculturable state of Pseudomonas aeruginosa induced by chlorine stress. Environ. Res..

[22] Guo L., Ye C., Cui L., Wan K., Chen S., Zhang S., Yu X. (2019). Population and single cell metabolic activity of UV-induced VBNC bacteria determined by CTC-FCM and DO-labeled Raman spectroscopy. Environ. Int..

[23] Brenner S. (1974). The genetics of Caenorhabditis elegans. Genetics.

[24] Li L., Fu J., Bae S. (2022). Changes in physiological states of Salmonella Typhimurium measured by qPCR with PMA and DyeTox13 Green Azide after pasteurization and UV treatment. Appl. Microbiol. Biotechnol..

[25] Chiang E.L.C., Lee S., Medriano C.A., Li L., Bae S. (2022). Assessment of physiological responses of bacteria to chlorine and UV disinfection using a plate count method, flow cytometry and viability PCR. J. Appl. Microbiol..

[26] Ding N., Liu K., Jiang L., Liu H. (2023). The temperature-dependent kinetics and bacteria regrowth by performic acid and sodium hypochlorite disinfection. Water Sci. Technol. J. Int. Assoc. Water Pollut. Res..

[27] Meng L., Ma J., Liu C., Mao X., Li J. (2022). The microbial stress responses of *Escherichia coli* and Staphylococcus aureus induced by chitooligosaccharide. Carbohydr. Polym..

[28] Yang D., Wang W., Zhao L., Rao L., Liao X. (2023). Resuscitation of viable but nonculturable bacteria promoted by ATP-mediated NAD+ synthesis. J. Adv. Res..

[29] Pan H., Yang D., Wang Y., Rao L., Liao X. (2023). Acid shock protein Asr induces protein aggregation to promote *E. coli O*157:H7 entering viable but non-culturable state under high pressure carbon dioxide stress. Food Microbiol..

[30] Borisov V.B., Siletsky S.A., Nastasi M.R., Forte E. (2021). ROS Defense Systems and Terminal Oxidases in Bacteria. Antioxidants.

[31] Ma Z., Xu W., Li S., Chen S., Yang Y., Li Z., Xing T., Zhao Z., Hou D., Li Q. (2024). Effect of RpoS on the survival, induction, resuscitation, morphology, and gene expression of viable but non-culturable Salmonella Enteritidis in powdered infant formula. Int. J. Food Microbiol..

[32] Masmoudi S., Denis M., Maalej S. (2010). Inactivation of the gene katA or sodA affects the transient entry into the viable but non-culturable response of Staphylococcus aureus in natural seawater at low temperature. Mar. Pollut. Bull..

[33] Zhao F., Bi X., Hao Y., Liao X. (2013). Induction of viable but nonculturable *Escherichia coli* O157:H7 by high pressure CO_2_ and its characteristics. PLoS ONE.

[34] Wei C., Zhao X. (2018). Induction of Viable but Nonculturable *Escherichia coli* O157:H7 by Low Temperature and Its Resuscitation. Front. Microbiol..

[35] Chaiyanan S., Chaiyanan S., Grim C., Maugel T., Huq A., Colwell R.R. (2007). Ultrastructure of coccoid viable but non-culturable *Vibrio cholerae*. Environ. Microbiol..

[36] Bai H., Zhao F., Li M., Qin L., Yu H., Lu L., Zhang T. (2019). Citric acid can force Staphylococcus aureus into viable but nonculturable state and its characteristics. Int. J. Food Microbiol..

[37] Hamabata T., Senoh M., Iwaki M., Nishiyama A., Yamamoto A., Shibayama K. (2021). Induction and Resuscitation of Viable but Nonculturable Corynebacterium diphtheriae. Microorganisms.

[38] Kong I.S., Bates T.C., Hülsmann A., Hassan H., Smith B.E., Oliver J.D. (2004). Role of catalase and oxyR in the viable but nonculturable state of Vibrio vulnificus. FEMS Microbiol. Ecol..

[39] Hu Z., Bai X. (2023). Self-repair and resuscitation of viable injured bacteria in chlorinated drinking water: Achromobacter as an example. Water Res..

