# Supplementary material for: Preliminary Study of the Characterization of the Viable but Noncultivable State of Yersinia enterocolitica Induced by Chloride and UV Irradiation
Source: Microorganisms. 2024 Aug 28;12(9):1778. doi: 10.3390/microorganisms12091778 (PMC11434376; doi:10.3390/microorganisms12091778)
Supplement: Supplementary file 1 [file microorganisms-12-01778-s001.zip › microorganisms-3174390-supplementary.pdf]

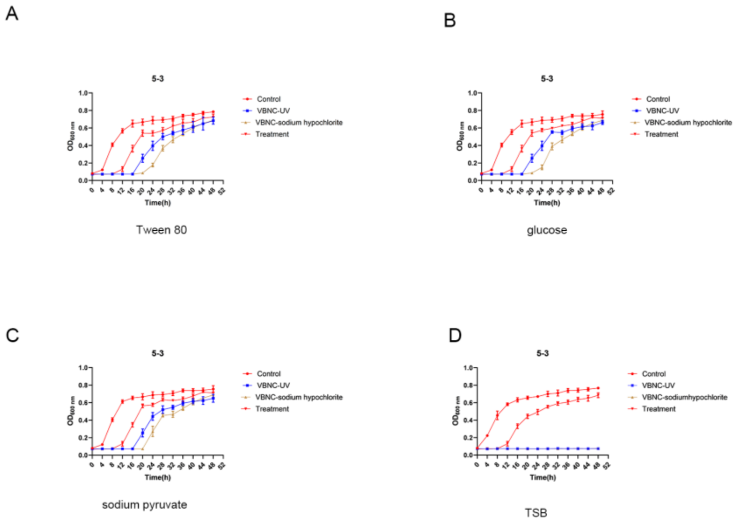

Figure S1:VBNC state *Y. enterocolitica* 5-3 induced by Naclo and UV recovery in Tween 80-TSB (A), glucose-TSB (B) and sodium pyruvate-TSB (C) and TSB(D).

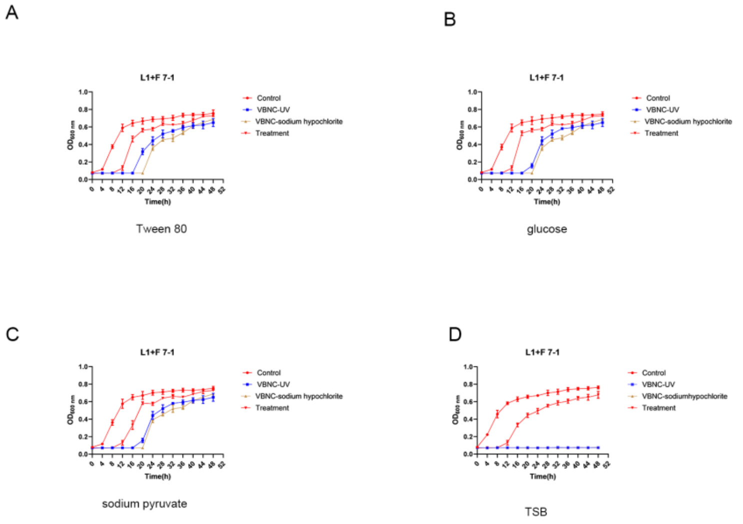

Figure S2:VBNC state *Y. enterocolitica* L1+F 7-1 induced by Naclo and UV recovery in Tween 80-TSB (A), glucose-TSB (B) and sodium pyruvate-TSB (C) and TSB(D).
